# Cystine/glutamate antiporter System x_c_^-^ deficiency impairs insulin secretion in mice

**DOI:** 10.1007/s00125-023-05993-6

**Published:** 2023-08-31

**Authors:** Axel de Baat, Daniel T. Meier, Leila Rachid, Adriano Fontana, Marianne Böni-Schnetzler, Marc Y. Donath

**Affiliations:** 1 Clinic of Endocrinology, Diabetes and Metabolism, University Hospital Basel, Basel, Switzerland; 2 Department of Biomedicine, University of Basel, Basel, Switzerland

**Keywords:** Beta cell, CHAC1, Cysteine, Cystine, Glutathione, Insulin, Islet, SLC7A11, System xc–

## Abstract

**Aims/hypothesis:**

Glutamate-induced cytotoxicity (excitotoxicity) has been detected in pancreatic beta cells. The cystine/glutamate antiporter System x_c_^-^ exports glutamate to the extracellular space and is therefore implicated as driving excitotoxicity. As of yet, it has not been investigated whether System x_c_^-^ contributes to pancreatic islet function.

**Methods:**

This study describes the implications of deficiency of System x_c_^-^ on glucose metabolism in both constitutive and myeloid cell-specific knockout mice using metabolic tests and diet-induced obesity. Pancreatic islets were isolated and analysed for beta cell function, glutathione levels and ER stress.

**Results:**

Constitutive System x_c_^-^ deficiency led to an approximately threefold decrease in glutathione levels in the pancreatic islets as well as cystine shortage characterised by upregulation of *Chac1*. This shortage further manifested as downregulation of beta cell identity genes and a tonic increase in endoplasmic reticulum stress markers, which resulted in diminished insulin secretion both in vitro and in vivo. Myeloid-specific deletion did not have a significant impact on metabolism or islet function.

**Conclusions/interpretation:**

These findings suggest that System x_c_^-^ is required for glutathione maintenance and insulin production in beta cells and that the system is dispensable for islet macrophage function.

**Graphical Abstract:**

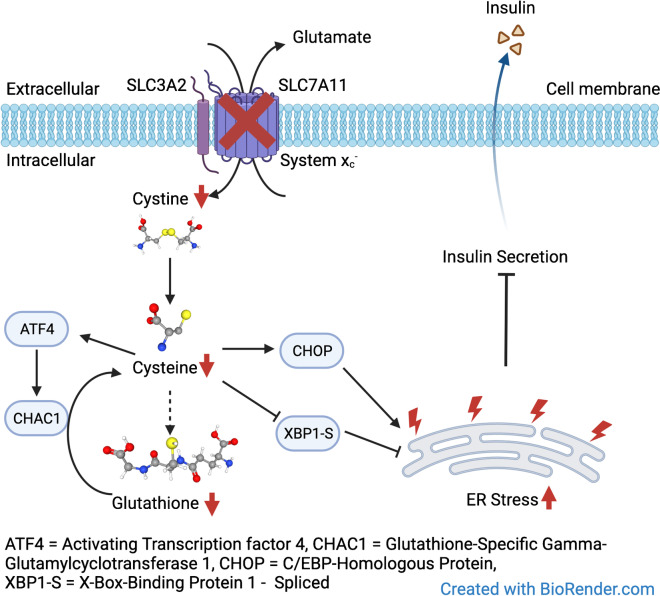

**Supplementary Information:**

The online version of this article (10.1007/s00125-023-05993-6) contains peer-reviewed but unedited supplementary material.

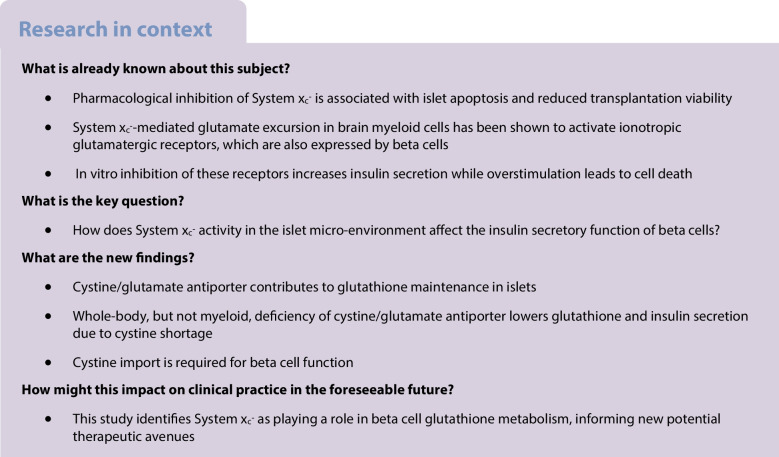



## Introduction

Insulin secretion in pancreatic beta cells is coupled to the many by-products of beta cell glucose catabolism and subsequent oxidative phosphorylation, ranging from ATP and glutamate [1] to reactive oxygen species (ROS) [2]. This relatively unrestricted flux of glucose metabolism makes beta cells especially sensitive to redox stress induced by hyperglycaemia [3, 4]. One of the main drivers of type 2 diabetes-associated cellular dysfunction is excessive production and defective detoxification of ROS [5]. Detoxification of ROS is performed by, among others, glutathione (GSH) peroxidases that require the thiol-containing tripeptide GSH as a co-factor [6]. The limiting substrate for the synthesis of GSH is cysteine, a semi-essential proteinogenic amino acid containing a thiol group that is utilised for redox chemistry [7]. While cysteine can be taken up by a range of amino acid transporters, its oxidised dimeric form cystine is mainly taken up through System x_c_^-^ (Sxc). Sxc is a heteromeric amino acid transporter comprised of a heavy chain (CD98), which it shares with many other amino acid transporters, and a unique light chain encoded by *Slc7a11*. The transporter functions as an antiporter, exchanging cystine for glutamate in a 1:1 ratio in a sodium-independent fashion [8]. *Slc7a11* is upregulated upon cystine starvation by activating transcription factor 4 (ATF4), in a mechanism whereby *ATF4* is preferentially transcribed when regular transcription slows down [9]. Both amino acid starvation and abnormal GSH levels result in endoplasmic reticulum (ER) stress, as the ER requires a steady rate of protein synthesis and a specific reducing environment for proper function [10]. Sxc inhibition with erastin leads to a type of iron-mediated cell death called ferroptosis, a process that has also been suggested to occur and impair islet viability during transplantation [11]. When cells are low on cysteine, GSH-specific γ-glutamyl cyclotransferase 1 (CHAC1, encoded by *CHAC1*) is upregulated. CHAC1 degrades GSH, thereby releasing cysteine from the tripeptide [12].

Type 2 diabetes is associated with islet amyloid formation followed by macrophage infiltration and activation in the pancreatic islets [13, 14]. During this process, profound metabolic and transcriptomic changes within the cell drive an anabolic response [15]. This response includes upregulation of *Slc7a11* to supply enough cysteine for the inflammatory response and to mitigate the associated increased ROS production [16].

Glutamate export by myeloid cells through Sxc has been implicated as an important factor in excitotoxicity, a neuronal cell death that is characterised by excessive glutamate-induced depolarisation [17]. Beta cells and neurons share developmental transcription factors, evolutionary origins and the utilisation of membrane depolarisation to release secretory granules [18]. It has previously been described that beta cells are sensitive to glutamate-induced cytotoxicity [19, 20]. Given the high sensitivity of beta cells to redox stress and the possible occurrence of excitotoxicity, we hypothesised that myeloid Sxc activity plays a critical role in the aforementioned processes. To test this hypothesis, as well as the impact of Sxc deficiency in beta cells, we used constitutive and conditional genetic mouse models of *Slc7a11* deficiency and studied consequences on metabolism in vivo and in vitro.

## Methods

### Mouse models

All animal experiments were conducted according to Swiss Veterinary Law and Institutional Guidelines and were approved by the Swiss Authorities. Mice were held in the vivarium at University Hospital Basel and were maintained under a 12 h dark–light cycle (starting at 06:00 hours) at 25°C. Cages were populated with two to six mice. Mice were given plastic housing and tissues as cage enrichment. All mice were specific-pathogen free. Mice were fed normal chow obtained from Granovit (Switzerland). The high-fat diet (HFD) was lard-based and obtained from Ssniff (Germany).

Constitutive *Slc7a11*-knockout (KO) mice were first generated by Sato et al [21]. The transgene was propagated by crossing heterozygous parents to ensure littermate representation in both experimental groups.

**Fig. 1:**
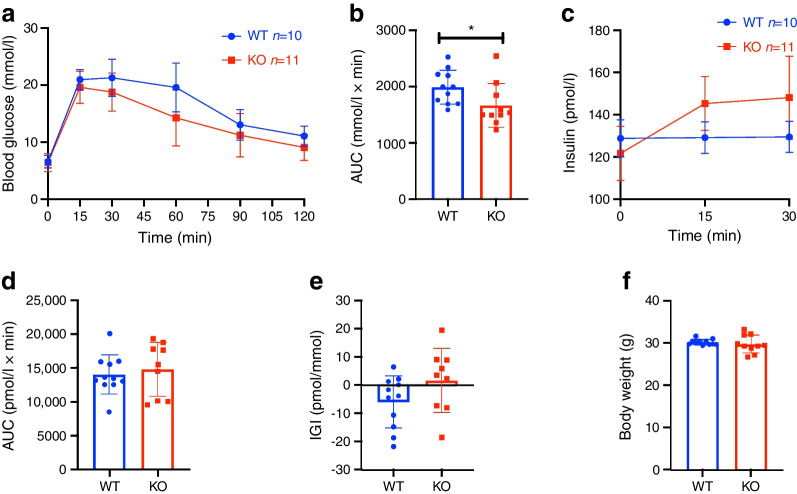
*Slc7a11*-KO increases insulin secretion and glucose disposal in 12-week-old mice. IPGTT blood glucose (**a**) with corresponding AUC (**b**), and plasma insulin (**c**) with corresponding AUC (**d**) in 12-week-old Sxc whole-body KO mice after i.p. injection of 2 g/kg glucose following a 6 h fast. (**e**,** f**) Insulinogenic index (**e**) and body weight (**f**) of the mice. Data are presented as mean ± SD. *p<0.05 (two-sided Mann–Whitney *U* test). IGI, insulinogenic index

The Institut Clinique de la Souris (ICS) (Illkirch-Graffenstaden, France) provided the mutant mouse line (allele: *Slc7a11*^tm1a(EUCOMM)Wtsi^), and INFRAFRONTIER/EMMA (www.infrafrontier.eu, PMID: 25414328) and PHENOMIN-TAAM (Orléans, France) distributed the mouse line (EM:10001). To generate the tm1b allele, mice were first crossed with B6-Tg(ACTFLPe)9205Dym/NPg (The Jackson Laboratory, USA, https://www.jax.org/strain/003800 backcrossed to our in-house C57Bl/6N background) to induce flipase-mediated recombination of FRT-flanked resistance cassette. To generate a conditional myeloid cell-specific *Slc7a11-* KO, floxed mice were crossed to B6N.129P2-*Lyz2*^tm1(cre)Ifo^ (The Jackson Laboratory, USA, https://www.jax.org/strain/004781 backcrossed to our in-house C57Bl/6N background). Mice carrying only flx were denoted as ‘Flx’ whereas mice carrying both flx alleles and a single copy of Cre-recombinase were denoted as ‘Cre’ (see electronic supplementary material (ESM) Fig. 1). The resulting mouse line did not exhibit any observable developmental defects. Knockdown efficiency was assessed by quantitative PCR.

Experiments were blinded and groups were randomised according to litter distribution of genotypes. Mice were bred so that all experimental groups contained representatives of a single litter. Mice were scored according to appearance, breathing, eyes, nose, behaviour, locomotion and body weight, humane endpoints were administered when a threshold score was reached.

### Endotoxaemia

To induce endotoxaemia, mice were injected with 2 mg/kg body weight phenol-extracted lipopolysaccharide (LPS; *Escherichia coli* O26:B6, cat. L8274, Sigma-Aldrich, Germany) dissolved in PBS. Mice were monitored regularly during the 6 h before they were euthanised or underwent metabolic experiments.

### IPGTT and IPITT

For the IPGTT, mice were fasted for 6 h (from 08:00 to 14:00 hours) and then injected with 2 g/kg body weight glucose (40% glucose solution wt/vol). To minimise stress, mice were moved to the experimental room 2 h before the experiment. Blood glucose was monitored at 0, 15, 30, 60, 90 and 120 min post injection using a Freestyle Lite glucometer (Abbott, Switzerland). Additionally, at 0, 15 and 30 min, 25 μl of blood was collected in tubes containing 2.5 μl of 50 mmol/l EDTA solution to obtain plasma for insulin measurements. Mice that displayed diarrhoea or leakage of the glucose solution from the injection site were excluded from the experiments.

Insulin resistance was assessed by ITTs in which mice were injected with 1 (1.5 for high-fat diet-fed mice) U/kg body weight Actrapid (Novo Nordisk, Denmark). Glucose was measured at 0, 15, 30, 60, 90 and 120 min post injection using a Freestyle Lite glucometer (Abbott, Switzerland). Glycaemic index was calculated as: (insulin at *t*=15 − insulin at *t*=0)/(blood glucose at *t*=15 − blood glucose at *t*=0), where t is the time point in minutes.

### 2-Deoxy[^3^H]glucose uptake and detection

To assess tissue glucose uptake, mice were fasted for 6 h and injected with 2 g/kg body weight glucose (40% glucose solution wt/vol) spiked with 350 μCi/kg body weight 2-deoxy[^3^H]glucose acquired from Perkin Elmers (cat. NET328001MC, USA). Tissues were harvested and 200 mg of the respective tissue was taken and homogenised in 500 μl ultrapure water with a stainless-steel bead in a TissueLyser II. The homogenates were then heated on a heating block at 95°C for 5 min and subsequently pelleted. Supernatant fractions were collected and phosphorylated 2-deoxy[^3^H]glucose was separated by Poly-Prep columns (cat. 7316212, BioRad, Germany). The final eluates were mixed with Ultima Gold Scintillation fluid (cat. L8411, Sigma Aldrich, Germany) at a 1:7 ratio in scintillation tubes and analysed with a beta counter.

### Adipose tissue isolation

Epididymal white adipose tissue was isolated and minced with scissors. The adipose tissue was then incubated with collagenase for 45 min at 37°C in a shaker. Stromal vascular fraction was obtained through centrifugation.

To isolate adipocytes, the digested adipose tissue was spun at 30 *g*, the infranatant fraction was removed and the floating fraction was washed and filtered through a 200 μm pore size mesh. Adipocytes were then distributed for experiments based on volume.

### Ex vivo adipocyte 2-deoxyglucose uptake

To assess glucose uptake in isolated adipocytes, a Promega glucose uptake glo assay (cat. J1341, Promega, Switzerland) based on 2-deoxy glucose was used. In short, isolated adipocytes were distributed, based on volume, in glass tubes in a 37°C shaker in modified Krebs-Ringer bicarbonate buffer (KRB: 115 mmol/lNaCl, 4.7 mmol/lKCl, 2.6 mmol/lCaCl_2_ 2H_2_O, 1.2 mmol/lKH_2_PO_4_, 1.2 mmol/lMgSO_4_ 7H_2_O, 10 mmol/lHEPES, 0.5% BSA, pH 7.4) containing 11 mmol/l glucose and 1 mmol/l 2-deoxyglucose with or without 100 nmol/l insulin. After shaking incubation for 1 h, the supernatant fraction was removed and cells were washed twice with KRB containing 11 mmol/l glucose. Cells were subsequently processed according to manufacturer’s protocol.

### Islet isolation

Islets were isolated by collagenase digestion via infusion of collagenase mix through the biliopancreatic duct. Islets were harvested by handpicking under a microscope and cultured in a cell culture incubator (37°C and 5% CO_2_) in DMEM containing 11.1 or 22.2 mmol/l glucose, 1 mmol/l pyruvate, non-essential amino acids, 100 U/ml penicillin, 100 μg/ml streptomycin, 2 mmol/l glutamine, 50 μg/ml gentamicin, 10 μg/ml fungison and 10% FCS, with or without 1 mmol/l cystine.

### Glucose-stimulated insulin secretion

The glucose-stimulated insulin secretion (GSIS) protocol was adapted from an earlier publication [22]. In short, islets were incubated in 96-well plates with one islet per well for 2–3 days in a cell culture incubator in DMEM. Islets were washed and then pre-incubated for 1 h in pre-equilibrated modified KRB containing 5.5 mmol/l glucose. This was followed by 1 h incubation to obtain basal secretion and then the medium was exchanged for 16.7 mmol/l glucose KRB to yield the stimulated sample. After the experiment, wells were checked by eye and any not containing an islet were excluded. Supernatant fractions from five wells were pooled per sample and three or four samples were taken per mouse. Insulin was quantified with the mesoscale mouse/rat insulin assay (cat. K152BZC, Mesoscale Discovery, USA). Stimulation index was calculated as the ratio of stimulated to basal secretion. γ-Glutamyl-cysteine (GGC) was kindly provided by Dr M. Zarka from Biospecialities (Australia).

### RNA isolation and quantitative real-time PCR

RNA was isolated by the Nucleo Spin RNA II Kit (cat. 740955, Macherey and Nagel, Germany). cDNA was prepared with the GoScript Reverse Transcription Mix (A2801, Promega) containing random primers according to the manufacturer’s instructions. For quantitative real-time PCR (qPCR) of *Chac1*, *Il1b* and *Slc7a11*, SYBRgreen-based chemistry with GoTaq Polymerase (cat. A6002 Promega) and the ABI 7500 fast System was employed (Applied Biosystems, USA). *Hprt* was used as a housekeeping gene and expression levels were calculated as (gene of interest − housekeeping gene) so as to not assume an amplification efficiency of 2 and to display the data in the most unadulterated manner. Primer sequences were all derived from the first ranked pairs in the Harvard Primer Bank (https://pga.mgh.harvard.edu/primerbank/, verified 24 July 2023).

### Flow cytometry

Flow cytometry experiments were performed on a CytoFLEX V2-B4-R2 Flow Cytometer (eight detectors, three lasers) (Beckman-Coulter, Germany). Antibodies used for surface staining are listed in ESM Table 1. For measuring intracellular thiol levels, ThiolTracker dye (T10095, Thermo Fisher Switzerland), was used according to the manufacturer’s directions. Cells were washed and subsequently analysed.

### Fluorescence-activated cell sorting of macrophages and dispersed islets fractions

After isolation, islets were dispersed and beta cells were sorted based on size and granularity as described before [23] and islet immune cells were sorted as the CD45^+^ fraction.

Macrophages from different tissues were identified as F4/80 - PE-Cy7 and CD11b - PerCP-Cy5.5 positive cells after being gated for singlet, DAPI-negative, lineage negative (CD19, Ly-6G, CD3, NK1.1 - BV510) accordingly. The cells were sorted on the FACSMelody obtained from BD (Switzerland).

### ATP and GSH measurements

ATP measurements were performed using CelltiterGlo3D (cat. G9681, Promega). For GSH measurements in islets, an adapted version of the glutathione-glo assay (cat. V6911, Promega, Switzerland) was performed with the standard lysis buffer being replaced with passive lysis buffer (cat. E1941, Promega, Switzerland) in order to prevent inadequate permeabilisation. One sample represents ten islets.

### Microscopy and beta cell mass quantification

The weight of the pancreas was determined at the time the mice were killed. Staining for insulin and CD45 was performed as previously described [24]. Pancreas and beta cell areas were determined using a convolutional neural network approach using the U-net architecture from https://github.com/jvanvugt/pytorch-unet (verified on 24 July 2023). The outputs were hand curated and analysed using ImageJ software (version 2.0.0-rc-68/152g, https://imagej.net/software/fiji/downloads). Normalised beta cell weight was calculated as a ratio: (beta cell area/pancreas area)×(pancreas weight/body weight).

### Data analysis and visualization

Results were analysed and plotted with Prism 9.1.0 (GraphPad, USA) or Python 3.9 (https://www.python.org/downloads/release/python-3913/).

### Statistics

*p*<0.05 was considered to be significant. Results were expressed as mean ± SD. Data were analysed with unpaired two-sided Mann–Whitney *U* test or two-way ANOVA. Statistical details of experiments are described in the figure legends.

## Results

### *Slc7a11*-deficient mice have increased insulin secretion and glucose disposal that is abrogated upon ageing and high-fat feeding

To understand the role of Sxc in glucose metabolism, we obtained constitutive *Slc7a11*-KO, generated by Sato et al through insertion of a selection cassette in exon 1 [21]. As previously described, no developmental alterations were observed in these mice. At 12 weeks of age, glucose disposal during an IPGTT was increased in the *Slc7a11*-KO mice (Fig. 1a, b), with a slight increase in insulin secretion that did not reach statistical significance (Fig. 1c–e). Body weight was comparable between the groups (Fig. 1f).

**Fig. 2:**
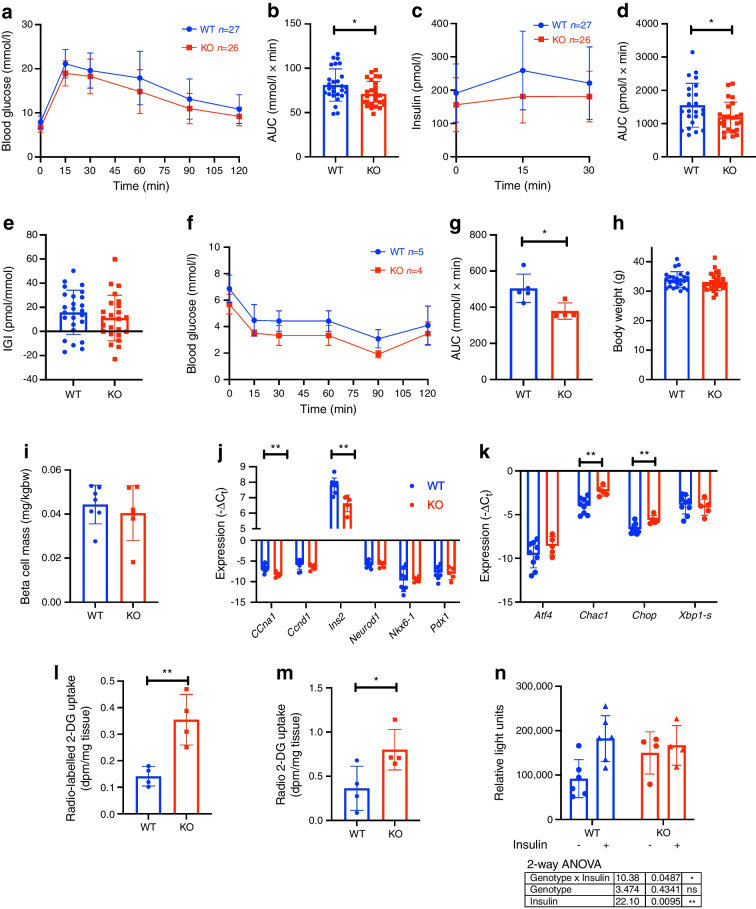
*Slc7a11*-deficient mice display increased glucose disposal and lower insulin secretion. (**a**–**d**) IPGTT blood glucose (**a**) with corresponding AUC (**b**), and plasma insulin (**c**) with corresponding AUC (**d**) in 26-week-old Sxc whole-body KO mice after i.p. injection of 2 g/kg glucose following a 6 h fast. (**e**–**h**) Insulinogenic index (**e**), IPITT blood glucose levels (**f**) with corresponding AUC (**g**), and body weight (**h**) of the mice. (**i**–**n**) Beta cell mass (**i**), expression of beta cell identity genes (**j**) and ER stress genes (**k**) in isolated islets, 2-deoxy[^3^H]glucose uptake in epididymal adipose tissue (**l**), and muscle (**m**) and glucose uptake in isolated adipocytes in response to 100 nmol/l insulin (**n**). Each data point represents one mouse. Data are presented as mean ± SD. *p<0.05 and **p<0.01 (two-sided Mann–Whitney *U* test, two-way ANOVA). bw, body weight; 2-DG, 2-deoxyglucose; IGI, insulinogenic index

At 26 weeks of age, increased glucose disposal was still apparent in the KO mice (Fig. 2a, b), although insulin secretion started to deteriorate compared with control mice (Fig. 2c–e). Glucose disposal after i.p. injection of insulin was increased in KO mice (Fig. 2f, g), although no difference in body weight was observed (Fig. 2h). To understand the underlying cause of the lower insulin secretion, beta cell mass was assessed by histology and found not to be different between genotypes (Fig. 2i). Expression levels of beta cell identity genes were screened in isolated islets (Fig. 2j). *Ccna1* (encoding cyclin A), a gene involved in beta cell proliferation, was significantly decreased in the KO mice; insulin expression, as assessed by *Ins2*, was also significantly decreased. We then screened genes associated with ER stress and found *Chac1* and *Chop* (also known as *Ddit3*) to be significantly upregulated (Fig. 2k), suggesting mild cysteine shortage and pro-apoptotic ER stress, respectively. Glucose uptake was then explored as an explanatory factor, by assessing radiolabelled glucose uptake. Glucose uptake after a non-labelled glucose bolus was increased in the epididymal adipose tissue and muscle of KO mice (Fig. 2l, m). Moreover in adipocytes isolated from Sxc-deficient mice, basal glucose uptake was increased but failed to be further enhanced in response to insulin (Fig. 2n). These data show a non-significant (*p* = 0.11) increase in insulinogenic index at 12 weeks that transitions to lower insulin secretion with mild beta cell ER stress in later life, compensated by increased glucose disposal.

**Fig. 3:**
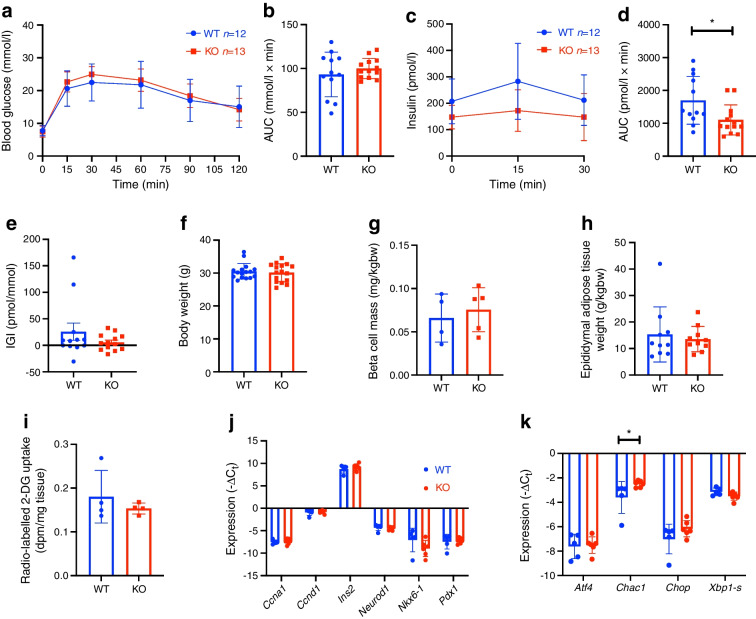
HFD feeding results in lower insulin secretion in mice. (**a**–**d**) IPGTT blood glucose (**a**) with corresponding AUC (**b**), and plasma insulin (**c**) with corresponding AUC (**d**) following i.p. injection of 2 g/kg glucose following a 6 h fast in 12-week-old whole-body male *Slc7a11*-deficient mice after 4 weeks of HFD feeding. (**e**–**h**) Insulinogenic index (**e**), body weight (**f**), beta cell mass (**g**), epididymal adipose tissue weight (**h**), 2-deoxy[^3^H]glucose uptake in epididymal adipose tissue (**i**), and expression profile of beta cell identity genes (**j**) and ER stress genes (**k**) in pancreatic islets isolated from the mice. Data are presented as mean ± SD. *p<0.05 (two-sided Mann–Whitney *U* test). bw, body weight; 2-DG, 2-deoxyglucose; IGI, insulinogenic index

Next, we investigated the implications of Sxc deficiency in high-energy diet feeding. Eight-week-old mice were fed HFD for 4 weeks. In contrast to mice on a chow diet, glucose disposal was not significantly different between genotypes (Fig. 3a, b). Similar to aged mice, insulin secretion was decreased in KO mice (Fig. 3c, d), while the insulinogenic index was unchanged (Fig. 3e). When comparing the genotypes, there was no difference in body weight (Fig. 3f) or beta cell mass (Fig. 3g). Additionally, epididymal adipose tissue weight and glucose uptake were similar between genotypes (Fig. 3h,i). Finally, we analysed the expression of beta cell identity genes in FACS-sorted beta cell-enriched fractions, finding no change in gene expression levels when comparing the two genotypes (Fig. 3j). We then also assessed ER stress genes and found only *Chac1* to be elevated (Fig. 3k). These experiments suggest that Sxc modestly increases insulin secretion and enhances glucose disposal in mice on a chow diet initially, while these effects disappear during obesity. This might be related to ER stress, as KO mouse islets show a modest increase in *Chac1* expression but no difference in beta cell identity genes.

### Sxc is required to maintain islet GSH levels and GSIS

**Fig. 4:**
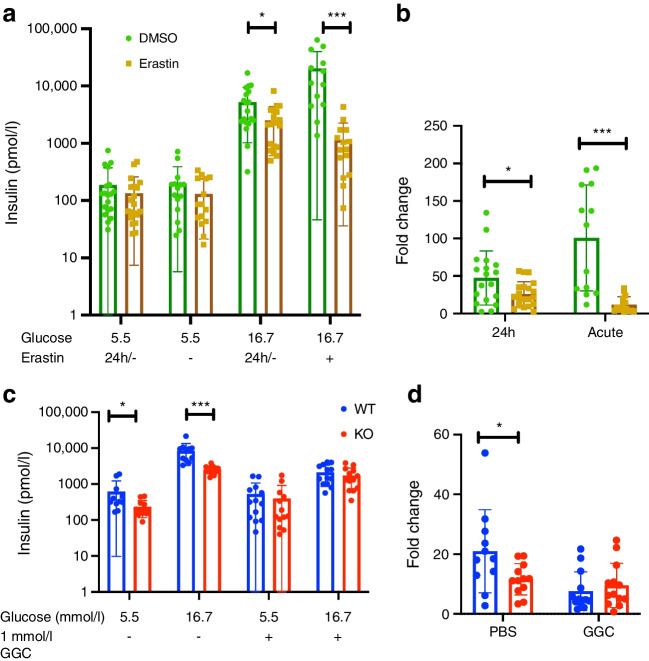
Effect of the Sxc inhibitor erastin (5 μmol/l) on insulin secretion in islets isolated from mice. (**a**) GSIS of islets treated with 5 μmol/l erastin for 24 h (24h/-), and GSIS of islets exposed to 5 μmol/l erastin only in medium containing 16.7 mmol/l glucose (- & +). **(b)** Corresponding fold change after 24 h and treatment only in high glucose medium. (**c**, **d**) GSIS of WT and KO mouse islets after treatment with 1 mmol/l GGC for 24 h (**c**), with the corresponding fold change **(d**). Each data point represents a pooled sample of five islets; three mice were used per group; data are the combined results of three experiments. Data are presented as mean ± SD. *p<0.05 and ***p<0.001 (two-sided Mann–Whitney *U* test)

To understand the effects of short term Sxc inhibition on acute insulin secretion, we used the Sxc inhibitor erastin. Exposure of islets isolated from wild-type (WT) mice to erastin for 24 h lowered GSIS (Fig. 4a, b). To investigate the effect of acute inhibition of Sxc on the GSIS, erastin was added only to medium containing 16.7 mmol/l glucose. When erastin was added only to the high-glucose stimulation medium, insulin secretion was almost completely abolished (Fig. 4a, b). Further, islets isolated from Sxc-deficient mice also showed diminished GSIS at both basal and glucose-stimulated conditions (Fig. 4c, d). We then wondered whether supplying GSH would rescue the impaired secretory phenotype. Pre-treatment of islets isolated from *Slc7a11*-KO mice and control mice with the GSH precursor γ-glutamyl-cysteine (GGC) removed the previously observed difference between the groups. However, GGC treatment also reduced the amount of insulin secreted in islets from both genotypes (Fig. 4c, d).

**Fig. 5:**
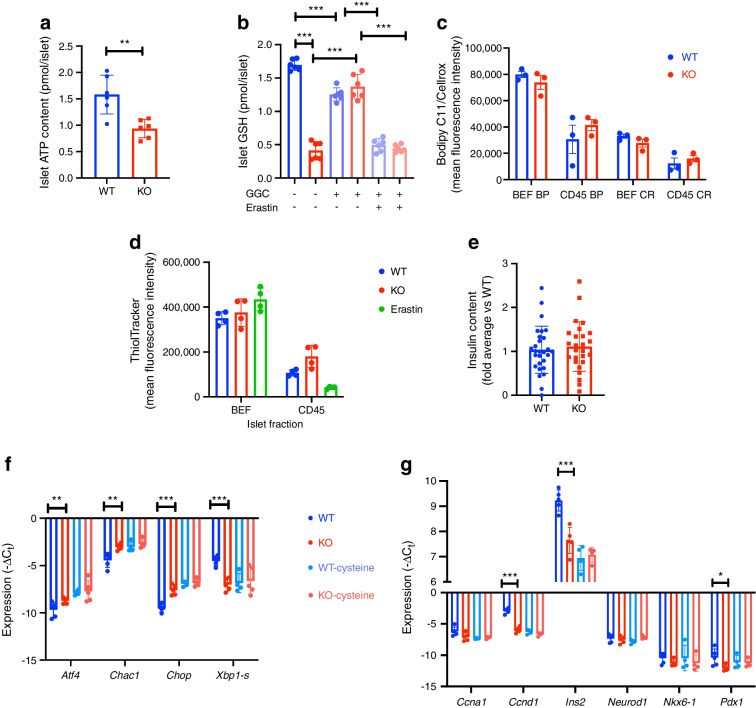
Suboptimal beta cell function in mice is driven by cystine shortage. (**a**, **b**) ATP levels (**a**) and GSH concentration (**b**) in whole islets from *Slc7a11-*KO and WT mice. (**c**) Lipid peroxidation and ROS measured by flow cytometry with Bodipy C11 and Cellrox in beta cell-enriched fraction and CD45^+^ fraction. (**d**) ThiolTracker thiol staining in dispersed islet fractions. (**e**–**g**) Insulin content (**e**) and expression levels of ER stress genes (**f**) and beta cell identity genes (**g**) in isolated islets. Data are presented as mean ± SD. (**a, b, e**) Each data point represents a ten-islet sample from one unique mouse. (**c**, **d**, **f**, **g**) Each data point represents one mouse. *p<0.05, **p<0.01 and ***p<0.001 (two-sided Mann–Whitney *U* test). BEF, beta cell-enriched fraction; BP, Bodipy C11; CR, Cellrox Green

To assess the ATP-generating potential in islets, as a proxy for mitochondrial function, ATP levels were analysed in isolated islets. The ATP content of islets from Sxc-KO mice was significantly lower than that of islets isolated from WT littermate control mice (Fig. 5a). We then determined GSH levels in whole islets and also found them to be significantly lower in islets from KO mice (Fig. 5b). GGC treatment partially prevented this decrease of GSH in KO islets but also reduced GSH levels in WT mouse islets to a level comparable with that in KO mouse islets given the same treatment. An additional group of islets was treated with erastin, which also lowered the GSH levels in KO mouse islets treated with GGC (Fig. 5b). This was surprising, since the islets lack Sxc, and suggests non-Sxc-mediated effects on GSH levels by erastin. Given the lower concentrations of GSH, an important co-factor for GSH peroxidases that scavenge hydrogen and organic and lipid peroxides, we next measured ROS and lipid peroxidation. Surprisingly, there was no difference between WT and KO mouse islet fractions (Fig. 5c), suggesting that the lower glutathione levels were still adequate for maintaining redox homeostasis. These data support the hypothesis that ablation of Sxc lowers insulin secretion, which is likely not driven by excessive redox stress but could be the result of a mild cysteine shortage that further diminishes GSH levels through *Chac1*. Finally, we evaluated whether the lower GSH levels result in lower total thiol levels and found thiols in beta cells to be similar between KO and WT mouse beta cells treated with erastin (Fig. 5d). However, Sxc inhibition reduced thiol levels in the islet immune CD45^+^ cell fraction (Fig. 5d). Insulin content within the islets did not differ between size-matched WT and KO mouse islets (Fig. 5e). To confirm that the observed transcriptional profile of islets was driven mostly by cysteine shortage, islets were cultured in high glucose with and without cystine added. Indeed, cystine deprivation led to an increase in the ER stress profile, with a decrease in *Xbp1-s*, which is associated with the pro-survival ER stress response (Fig. 5f). When WT mouse islets were deprived of cysteine, they showed a comparable ER stress response to that shown by KO mouse islets. High-glucose treatment precipitated a decrease in beta cell markers *Ccnd1*, *Ins2* and *Pdx1* in the KO mouse islets compared with WT mouse islets whereas depletion of cystine again resulted in similar transcriptional profiles in WT and KO mouse islets (Fig. 5g). These data suggest that cysteine shortage drives suboptimal beta cell function in Sxc deficiency.

### In vivo LPS treatment does not lead to a differential response when comparing Sxc-deficient and WT mice

Because LPS exposure leads to rapid upregulation of *Slc7a11* [16], we hypothesised that LPS pre-treatment might precipitate Sxc’s role in metabolism. We therefore injected whole-body Sxc-deficient mice with LPS (2 mg/kg body weight) 6 h before an IPGTT. We confirmed the previously reported increase in insulin secretion and glucose disposal [25] but failed to detect a differential response between WT and KO mice (ESM Fig. 2a–c). Additionally, plasma IL-1β levels were similar between genotypes (ESM Fig. 2d). Whole-blood GSH levels showed no differential response to LPS administration when comparing the genotypes (ESM Fig. 2e).

### Myeloid-specific Sxc deficiency does not phenocopy whole-body KO mice

**Fig. 6:**
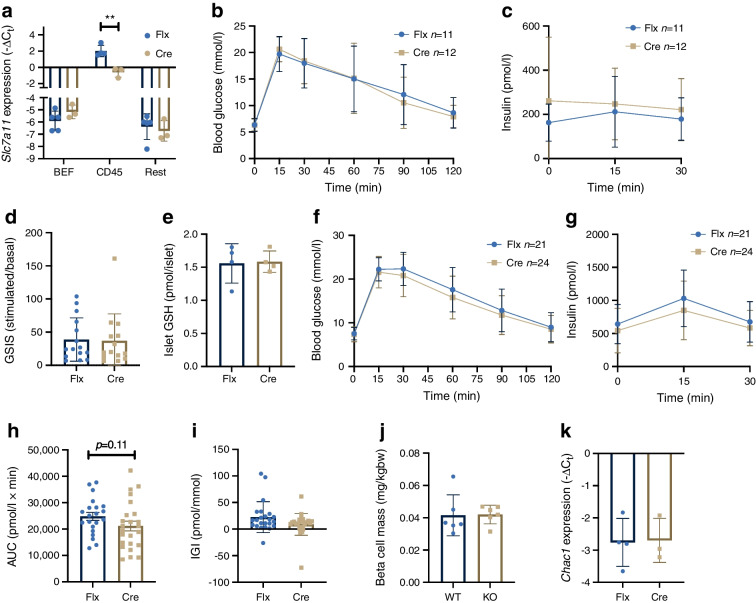
Myeloid-specific *Slc7a11* deficiency does not affect mouse beta cell function. (**a**) qPCR analysis of expression of *Slc7a11* in sorted islet fractions from Flx vs Cre mice. (**b**,** c**) IPGTT blood glucose (**b**) and plasma insulin levels (**c**) after i.p. injection of mice with 2 g/kg glucose following a 6 h fast. (**d**) GSIS of isolated islets. (**e**) GSH levels in whole islets normalised per islet. (**f**–**k**) IPGTT blood glucose (**f**), plasma insulin levels (**g**) with corresponding AUC (**h**) after i.p. injection of 2 g/kg glucose following a 6h fast, insulinogenic index (**i**), beta cell mass (**j**), and qPCR for *Chac1* in sorted beta cell-enriched fraction (**k**) in Flx and Cre mice fed HFD for 4 weeks. In (**d**), Each data point represents a pooled sample of five islets, three mice were used per experiment. In (**e**), one data point represents a ten-islet sample from a unique mouse. Data are presented as mean ± SD. **p<0.01 (two-sided Mann–Whitney *U* test). BEF, beta cell-enriched fraction; bw, body weight; IGI, insulinogenic index

Macrophages within islets have previously been shown to be involved in the regulation of insulin secretion [26]. Furthermore, *Slc7a11* expression levels in the immune cell fraction was 225-fold higher compared with other islet cell fractions (Fig. 6a). We therefore crossed mice carrying the *Slc7a11* gene with exon 3 flanked by loxP sites to *Lyz2*-Cre mice to obtain myeloid cell-specific *Slc7a11*-KO mice (ESM Fig. 1). *Slc7a11* expression was reduced by 83±9% (mean ± SD) in islet immune cells, showing efficient gene knockdown in this model (Fig. 6a). The improved metabolic phenotype observed in whole-body Sxc-deficient mice at 26 weeks of age was not present in mice with a myeloid-specific deletion (Fig. 6b, c). Similarly, ex vivo, we did not find a significant difference in insulin secretion in these islets (Fig. 6d). In contrast to our findings in the whole-body KO mice, the myeloid cell-specific KO mice did not display a decrease in islet-wide GSH levels (Fig. 6e). Next, we fed myeloid Sxc-deficient mice an HFD for 4 weeks. Blood glucose following a glucose bolus was unchanged between the groups but insulin secretion was lower in conditional KO mice (although the difference did not reach statistical significance, AUC *p*=0.11) (Fig. 6f–i). Beta cell mass and *Chac1* expression in the beta cell-enriched fraction was similar between genotypes (Fig. 6j,k). In sorted adipose tissue and peritoneal macrophages from *Lyz2*-Cre *Slc7a11*-KO mice there was a non-significant increase in *Chac1* expression levels relative to WT mice (ESM Fig. 3e–g). Further, female *Lyz2*-Cre *Slc7a11*-KO mice fed an HFD for 4 or 12 weeks did not show altered insulin sensitivity or glucose metabolism (ESM Fig. 4). We additionally performed GTTs in mice fed HFD for 12 and 44 weeks and did not find a difference between the KO and WT mice (ESM Fig. 5). These data suggest that despite a high expression level of *Sxc* in islet macrophages relative to endocrine and other islet cells, constitutive myeloid cell-specific Sxc deficiency does not affect islet function.

## Discussion

In the present study, we explored the function of the cystine/glutamate transporter Sxc in vitro as well as in vivo in the context of beta cell and macrophage function.

At 12 weeks of age, Sxc-deficient mice have a mildly (20%) increased glucose disposal. At 26 weeks of age, the Sxc-deficient mice additionally develop mildly lower insulin secretion (28% AUC). This suggests that the observed insulin secretory phenotype is relatively subtle and takes longer to manifest. Additionally, there seems to be a compensatory increase in glucose disposal in response to lower insulin secretion, resulting in lower or unchanged blood glucose (13% AUC). This could be a compensatory response to constitutive lower insulin secretion, to increase insulin sensitivity, as supported by the increased insulin sensitivity measured by the ITT (25% AUC). This could also suggest that immune cells deficient in *Slc7a11* might not be able to induce insulin resistance as effectively, although the intrinsically lower insulin secretion does not support this hypothesis. The observed lower insulin secretion is not caused by lower beta cell mass, so there might be a reduction in functional secretory capacity of the beta cells or an innate increase in insulin sensitivity.

When assessing insulin secretion by ex vivo GSIS, we found lower insulin output in response to glucose in *Slc7a11*-KO mouse islets (45% in GSIS), corroborating our in vivo findings and supporting an islet intrinsic defect over a systemic adaptation. Addition of the GSH precursor GGC abolished the difference in insulin secretion between WT and KO mouse islets. It has to be noted, however, that overall insulin output was significantly decreased in both groups by the treatment. This could be due to the final step in GSH synthesis, where glutamate is condensed with GGC, an ATP-dependent process that might consume ATP, which is critical for GSIS.

Lower GSH levels could also contribute to a lack of redox buffering capacity within the cell, namely through GSH’s role as co-factor in ROS-detoxifying enzymes. We were not able to confirm this hypothesis using flow cytometry-based redox measurements in dispersed islets. However, these dyes are not very sensitive, so it is possible that Sxc-deficient beta cells experience low-grade redox stress that can be compensated for and that is too subtle to measure with the currently employed methods. Further, lower GSH levels might also affect mitochondrial energy generation. Indeed, we found lower levels of ATP in the islets, and this could either be attributed to lowered cell viability or to decreased mitochondrial activity.

The differential glucose metabolism observed in chow-fed mice disappeared when mice were fed an HFD. *Slc7a11*-KO mice in this setting were not able to increase their insulin secretion to levels similar to those seen in WT mice (35% decrease in AUC). Despite the islet expansion induced by HFD and lower insulin secretion in response to a glucose bolus, there was no difference in beta cell mass between genotypes, again indicating a functional defect not related to beta cell maintenance or expansion.

Islets from Sxc-deficient mice showed lower transcriptional levels of beta cell identity genes such as *Ins2* and *Pdx1* indicating suboptimal beta cell function as well as decreased expression of cyclins associated with beta cell proliferation. Cystine deprivation produced a transcriptional profile in WT mouse islets similar to that in KO mouse islets cultured with cystine, suggesting cystine shortage is a driving factor.

When we sought to monitor the effect of Sxc deficiency on thiol levels, we found no difference in total thiol levels in islet cell fractions. Despite the lower GSH levels, the thiol levels in islets were comparable between genotypes. This suggests that, despite lower GSH, the total thiol levels of Sxc-deficient islet cells are similar between genotypes. Lacking the transporter could be compensated for by higher thiol retention in islet cells or compensatory upregulation of other pathways through which the cell can acquire sulphur-containing metabolites.

The high *Slc7a11* expression levels of islet immune cells compared with other islet fractions prompted us to study the myeloid-specific role of *Slc7a11* in islet function. While the mice were observed in many different settings, no metabolic effect of myeloid-specific Sxc deficiency was observed. This is of importance, because excessive glutamate (as observed in beta cell lines exposed to high-glucose concentrations) promotes apoptosis, which is mediated by *N*-methyl-d-aspartate (NMDA) receptor signalling [19]. NMDA receptor inhibition has also been shown to increase the depolarisation plateaus observed during insulin secretion, leading to higher insulin output and more efficient glucose disposal. Furthermore, Sxc in astrocytes and microglia has been implicated as the main culprit in glutamate-induced neurotoxicity [17, 27, 28]. We were not able to find an improvement in islet function upon myeloid Sxc deficiency. These data therefore suggest that myeloid Sxc does not contribute to glutamate-induced excitotoxicity of beta cells.

Concluding, Sxc whole-body deficiency leads to impaired insulin secretion and a mild cysteine shortage as evidenced by *Chac1* upregulation. Myeloid-specific Sxc deficiency does not affect insulin secretion or cysteine shortage, and does not protect against HFD-induced islet dysfunction.

### Supplementary Information

Below is the link to the electronic supplementary material.

Supplementary file1 (PDF 391 KB)

## Data Availability

Data are available from the authors upon reasonable request.

## Abbreviations

ATF4: Activating transcription factor 4
CHAC1: GSH-specific γ-glutamyl cyclotransferase 1
ER: Endoplasmic reticulum
GGC: γ-Glutamyl-cysteine
GSH: Glutathione
GSIS: Glucose-stimulated insulin secretion
HFD: High-fat diet
KO: Knockout
KRB: Krebs-Ringer bicarbonate buffer
LPS: Lipopolysaccharide
NMDA: *N*-methyl-d-aspartate
qPCR: Quantitative real-time PCR
ROS: Reactive oxygen species
Sxc: System x_c_^-^
WT: Wild-type
