# Supplementary material for: Cystine/glutamate antiporter System xc- deficiency impairs insulin secretion in mice
Source: Diabetologia. 2023 Aug 31;66(11):2062–74. doi: 10.1007/s00125-023-05993-6 (PMC10541846; doi:10.1007/s00125-023-05993-6)
Supplement: Supplementary file 1 — Supplementary file1 (PDF 391 KB) [file 125_2023_5993_MOESM1_ESM.pdf]

ESM Table 1 Fluorescent antibodies used in the study

Target	Fluorochrome	Clone	Supplier	Cat. Nr
CD45	PE-Cy7	30-F11	Biolegend	103113
F4/80	PE-Cy7	BM8	Biolegend	123113
CD11b	PerCP-Cy5.5	M1/70	Biolegend	101227
CD19	BV510	6D5	Biolegend	115545
Ly-6G	BV510	1A8	Biolegend	127633
CD3	BV510	17A2	Biolegend	100233
NK1.1	BV510	PK136	Biolegend	108737

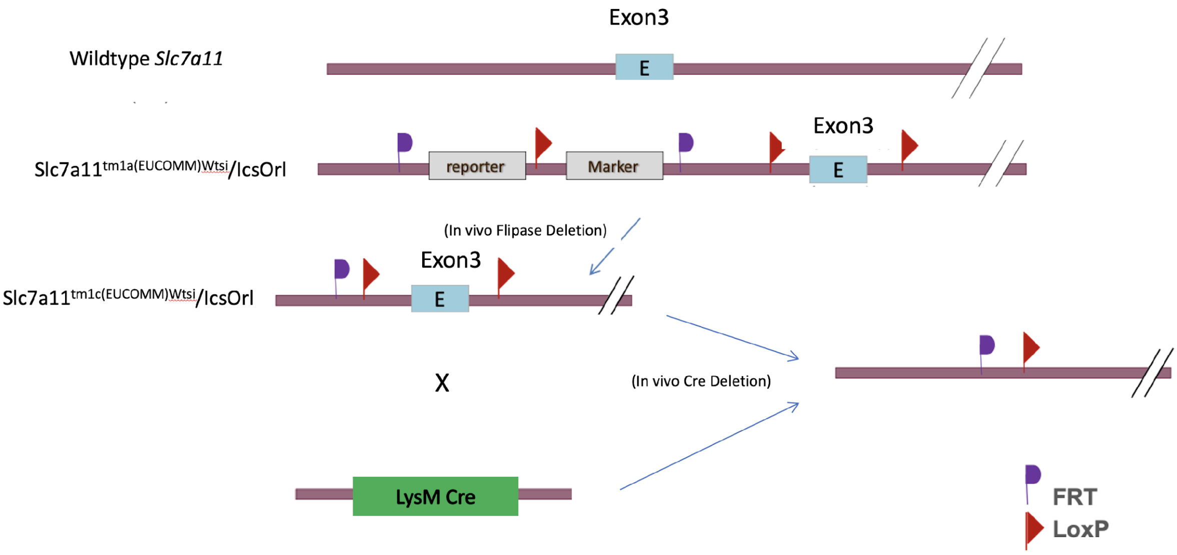

ESM Figure 1 Recombination strategy for generation of the myeloid-specific *Slc7a11* deficient animals. The mice carrying the tm1a allele were crossed with a flipase transgenic mouse to remove the reporter and marker cassette. Subsequently, by crossing homozygous tm1c carrying animals with a Cre-recombinase driven by a *Lyz2* promoter, myeloid-specific *Slc7a11* deficiency was established.

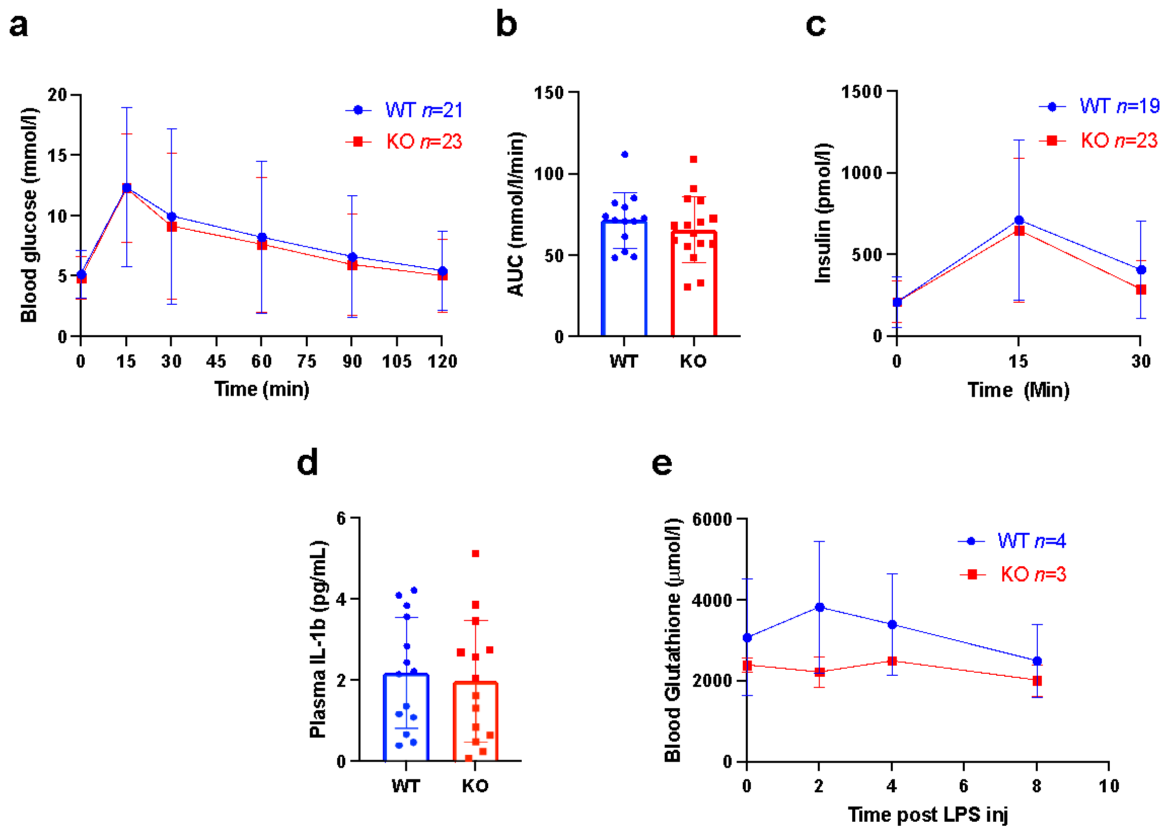

ESM Figure 2 IPGTT with LPS pre-treatment 6 hours before experiment in whole body *Slc7a11* deficient animals. (a) IPGTT glycemia, (b) corresponding AUC, (c) circulating insulin after intraperitoneal injection of 2g/kg glucose following a 6 hour fast. (d) Plasma IL-1b 6 hours post LPS injection, (e) whole blood glutathione time-course after 2mg/kgbw LPS injection. Error bars represent SD.

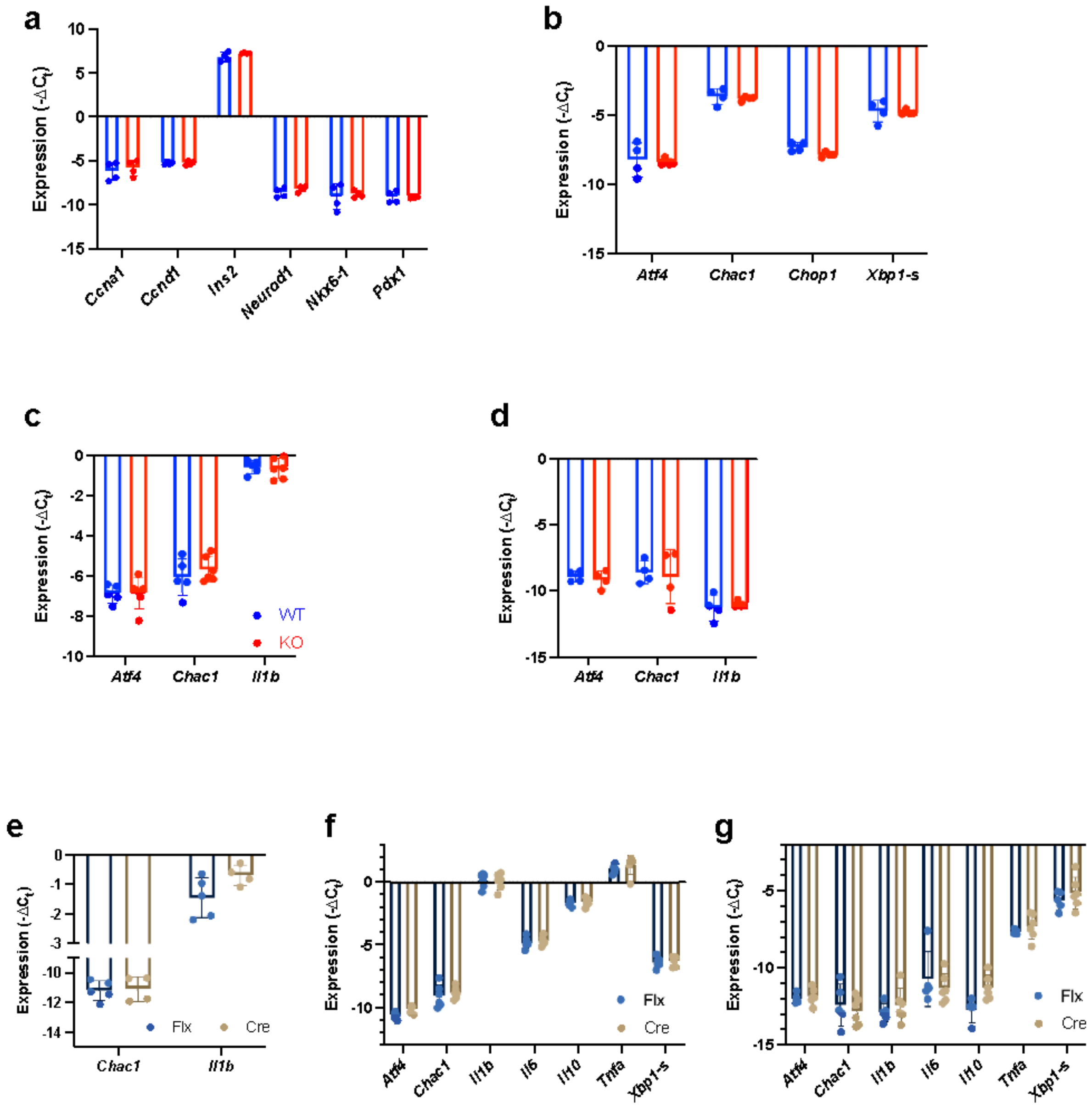

ESM Figure 3 qPCR screening of Erastin-treated islets isolated from 12-week-old whole-body KO mice (a,b) and sorted CD11b^{+} F480^{+} macrophages from adipose tissue and peritoneal lavage. (c,d) qPCR of peritoneal and adipose tissue macrophages isolated from 26-week-old whole-body KO mice. qPCR of ATMs (e,f) or peritoneal macrophages (g) isolated from myeloid-specific KO mice fed HFD for 4 or 24 weeks. Error bars represent SD.

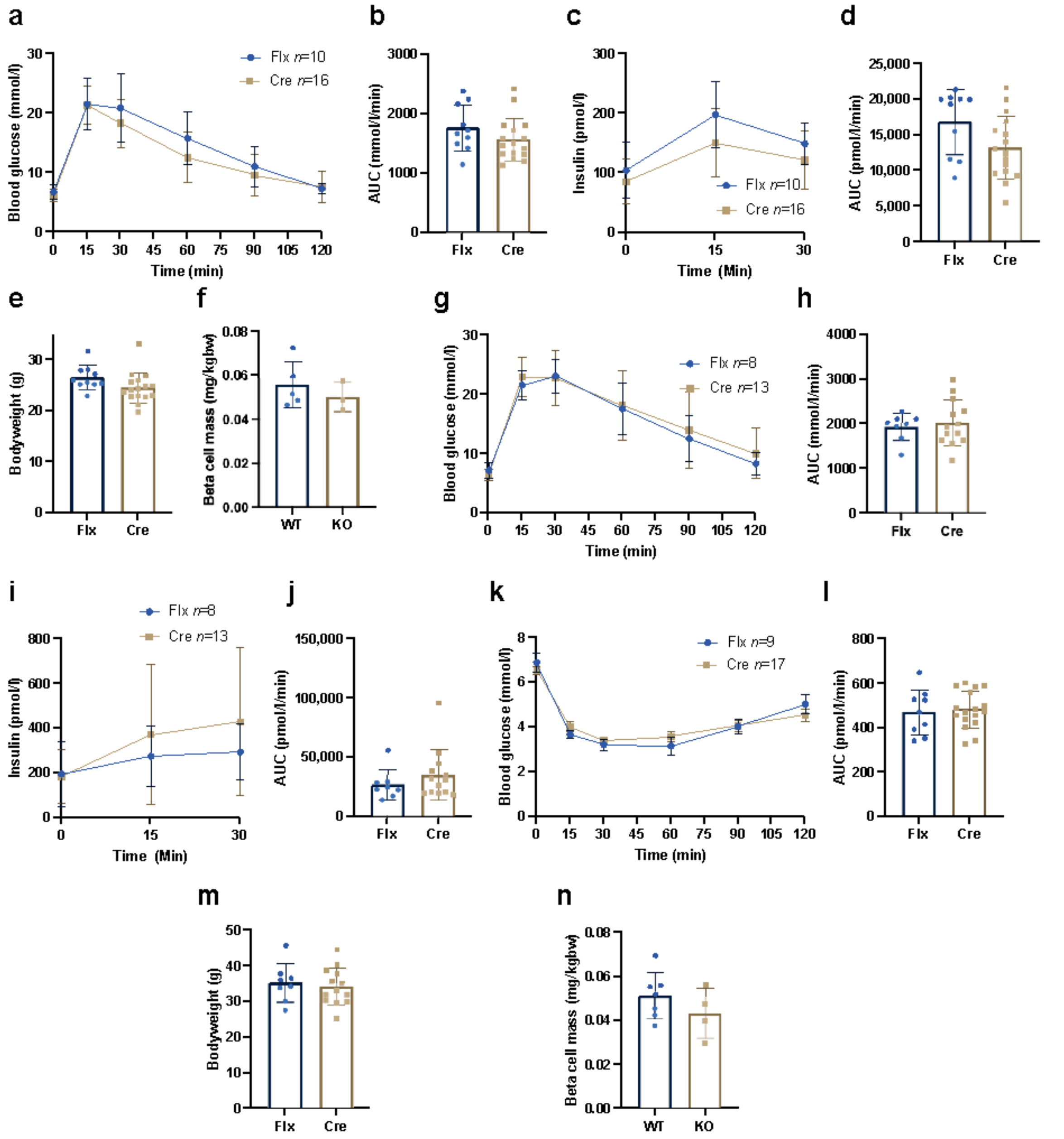

ESM Figure 4 LysM Cre *Slc7a11* Flx females metabolic experiments. (a-d) IPGTT, (e) body weight, (f) Beta-cell mass of mice fed HFD for 4 weeks.
(g-j) IPGTT, IPITT(k,l), Body weight of mice fed HFD for 12 weeks. (i-m), (n) Beta-cell mass of 8-week-old mice (before start HFD). Statistics: two-sided Mann–Whitney U test; Error bars represent SD.

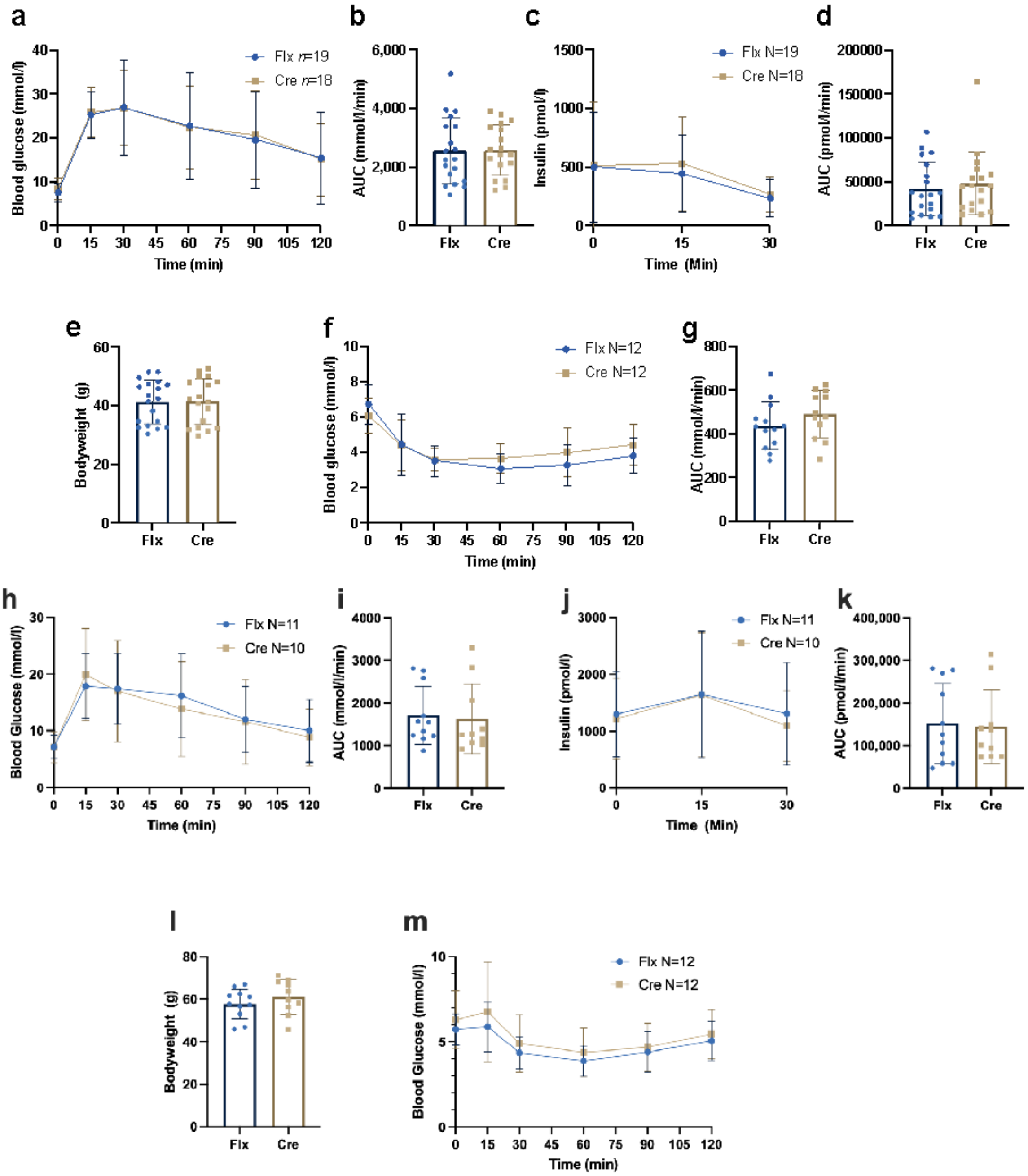

ESM Figure 5 Metabolic phenotype of male LysM Cre *Slc7a11* Flx mice fed HFD for 12 weeks. (a) ipGTT glycemia, (b) corresponding AUC, (c) circulating insulin and (d) corresponding AUC after intraperitoneal injection of 2g/kg glucose following a 6h fast and (e) body weight., (f,g) Insulin tolerance test. (h) ipGTT glycemia, (i) insulin, (j) AUC of glycemia, (k) AUC of insulin, (l) body weight, (m) insulin tolerance test of female LysM Cre *Slc7a11* Flx fed HFD for 44 week. Statistics: two-sided Mann–Whitney U test; Error bars represent SD.
